# Barriers and facilitators to implementing the HEADSS psychosocial screening tool for adolescents living with HIV/AIDS in teen club program in Malawi: health care providers perspectives

**DOI:** 10.1186/s13033-022-00520-3

**Published:** 2022-01-31

**Authors:** Esther C. Kip, Michael Udedi, Kazione Kulisewa, Vivian F. Go, Bradley N. Gaynes

**Affiliations:** 1 Malawi College of Medicine, Private Bag 360, Blantyre, Malawi; 2 Malawi Ministry of Health, Lilongwe, Malawi; 3 University of North Carolina, Chapel Hill, NC USA

**Keywords:** Adolescents, Adherence, Barriers, CFIR, HIV, Facilitators, Depression

## Abstract

**Background:**

Adolescents living with HIV (ALHIV) are at high risk of experiencing mental health problems. Depression is a major contributor to the burden of HIV-related disease amongst ALHIV and is significantly linked to non-adherence to anti-retroviral therapy (ART), yet it is under-recognized. In 2015, the Baylor College of Medicine International Pediatric AIDS Initiative (BIPAI) recommended that the psychosocial screening tool Home, Education, Activities, Drugs, Sexuality, Suicide/Depression (HEADSS) be used to screen ALHIV in Malawi who were part of an adolescent antiretroviral therapy program termed “Teen Club”. However, the HEADSS tool has been substantially under-utilized. This study assessed barriers and facilitators to implementing HEADSS for ALHIV attending Teen Club Program in four selected health facilities in Malawi.

**Methods:**

We conducted a qualitative study using semi-structured interviews at four program sites (one district hospital and one health center each in two districts) between April and May 2019. Twenty key informants were purposively selected to join this study based on their role and experiences. We used the five domains of the Consolidated Framework for Implementation Research (CFIR) to guide the development of the interview guides, analysis and interpretation of results.

**Results:**

Barriers included inadequate planning for integration of the HEADSS approach; concerns that the HEADSS tool was too long, time consuming, lacked appropriate cultural context, and increased workload; and reports by participants that they did not have knowledge and skills to screen ALHIV using this tool. Facilitators to implementing the screening were that health care providers viewed screening as a guide to better systematic counselling, believed that screening could build better client provider relationship, and thought that it could fit into the existing work practice since it is not complex.

**Conclusions:**

A culturally adapted screening tool, especially one that can be used by non-clinicians such as lay health workers, would improve the ability to address mental health needs of ALHIV in many primary care and social service settings where resources for professional mental health staff are limited. These findings are a springboard for efforts to culturally adapt the HEADSS screening tool for detection of mental and risky behaviors among ALHIV attending ART program in Malawi.

## Background

Globally, more than three million children are infected with human immunodeficiency virus (HIV), 90% of whom live in sub-Saharan Africa [1]. Now that the epidemic has matured and more children are accessing antiretroviral therapy (ART), children who were born with HIV are aging into adolescence in large numbers [1, 2]. Since the HIV services in sub-Saharan countries such as Malawi have successfully scaled up, improved survival rates of HIV-infected adolescents have raised awareness of the need for holistic care that consider mental health and quality-of-life issues [1–3].

Mental health disorders, such as high levels of anxiety, isolation, depression and suicide ideation, have been reported among ALHIV (10–24 years) and contribute significantly to poor medication adherence and retention in care [4–6]. Further, these mental health issues have been linked to increased sexual risk behavior [5, 6]. Depression, in particular, is a serious health problem among adolescents living with HIV (ALHIV), affecting both family and peer relationships, with suicide the most serious potential outcome [4–9]. In Malawi, a study of depression in ALHIV revealed a prevalence rate of 18.9% [1, 2, 4]. Depression is significantly linked to non-adherence to medication, making its diagnosis and treatment vital in the long-term survival of ALHIV [6, 8, 10]. Despite the high burden of depression and the availability of effective treatments, approximately 60% to 80% of affected adolescents do not receive appropriate care [10].

Child and adolescent primary care clinics are an important site for the identification of depression in adolescents, the critical first step in connecting youth to treatment [3, 8, 10, 11]. However, depression remains poorly identified in such settings [9, 10]. Research supports the screening of adolescents for depression in primary care [7, 9, 10]. Further, studies suggest that non-systematic approaches, such as primary care providers’ reliance on presenting complaints and family concerns to identify depressed youth, yields severe under-identification [9, 10]. Research has demonstrated that it is feasible to implement a depression screening and treatment program in primary care settings [7, 9–11]. Primary care settings can be ideal locations for screening for early detection of risky or dependent substance use, conducting brief interventions to reduce individual and population risk, initiating treatment for substance dependence, and managing other comorbid conditions that may benefit from specialist consultation and/or treatment [9–12].

### The HEADSS psychosocial screening

The Home, Education, Activities, Drugs, Sexuality, Suicide/Depression (HEADSS, see Table 1) psychosocial screening designed to improve identification of psychosocial issues, such as depression and high-risk behaviors in adolescents [13–21]. It has been validated in outpatient departments in the US and found to accurately screen for mental health problems in young people with reported sensitivity of 82% and a specificity of 87% in predicting psychiatric consult and admission to in-patient psychiatry [16, 17]. Efforts to implement it have also been successfully tested [15–18].

**Table 1:** HEADSS domains and interview questions

Domain	Examples of Questions
Home	Where do you live? Who do you live with? How much time do you spend at home? What do you and your family argue about? Can you go to your parents with problems? Have you ever run away from home?
Education and employment	What grade are you in? What grades are you getting? Have they changed? Have you ever failed any classes or been kept back a grade? Do you ever cut classes? Have you ever been teased or attacked at school? Do you work after school or on weekends? What are your career/vocational goals?
Activities	What do you do for fun? What activities do you do during and after school? Are you active in sports? Do you exercise? Who do you do fun things with? Who are your friends? Who do you go to with problems? What do you do on weekends? Evenings?
Drugs	Do you drink coffee or tea? Do you smoke cigarettes? Have you ever smoked one? Have you ever tried alcohol? When? What kind and how often? Do any of your friends drink or use drugs? What drugs have you tried? Have you ever injected steroids or drugs? When? How often do you use them? How do you get money to pay for drugs? Are drugs used or available in places where you hang out?
Sexual Activity/Identity	Do you feel you are ready for sex? Have you chosen to remain abstinent? Have you ever had sex? How many sexual partners have you had? How old were you when you first had sex? How old was your partner? Have you ever had sex with men? Women? Both? Do you think you might be lesbian, gay, or bisexual? Do you think you need to have sex to find out if you're lesbian, gay, or bisexual? Do you want to become pregnant? Have you ever been pregnant? Have you ever had an infection as a result of having sex? Do you use condoms or another form of contraception for STD and HIV prevention? Have you ever had sex unwillingly? Have you ever tried sex for money, drugs, clothes, or a place to stay? Have you ever been tested for HIV? Do you think it would be a good idea to be tested?
Suicide Risk/Depression Screening	How do you feel today, on a scale of 0—10 (0 = very sad, 10 = very happy)? Have you ever felt less than a 5? How long did that feeling last? What made you feel that way? Does thinking you may be lesbian, gay, or bisexual make you feel that way? Did you ever think about hurting yourself or that life isn't worth living, or hope that when you go to sleep you won't wake up?

Source: Adapted from Goldenring and Rosen [20]

This interview is a practical, time-tested strategy that physicians can use for psychosocial review of systems to evaluate how their teenaged patients are coping with the pressures of daily living including family, peers, school, culture and their inner world [12, 13, 20]. In addition, this screening tool is used all over the world to screen and identify high risk behaviors in adolescents. It also helps in guiding health counselling, including commending and building on strengths, exploring options, planning actions, providing information, identifying need for intervention and referral [17–21].

A study in Kenya using HEADSS newly identified 12 adolescents with suicidal ideation (4% of the study population) [16]. The researchers commented that this finding alone is a strong justification for routine use of HEADSS tool in assessing the ALHIV [16]. Another study in Ghana using HEADSS approach found a high psychosocial burden and a higher risk of mental health problems among ALHIV [21]. The factors identified with depression included unfavorable home situation, body image concerns, social isolation and legitimate HIV diagnosis –related fears such as stigma, dying young, and not marrying or having a sexual relationship [21]**.** In Tanzania, the National Adolescent Health and Development Strategy 2018–2022 recommended the HEADSS assessment for finding out issues among adolescents and providing necessary support which is usually not offered in facilities [22].

In Malawi, an ideal place to administer this screen are adolescent-specific ART clinics termed “Teen Clubs”, where Malawian ALHIV receive care and support. The teen-club model as a ‘one-stop center’ tries to cater for medical, social, relational, sexual reproductive (SRH) and psychosocial support [3, 8, 23–26]. It provides a space for ALHIV to walk in at any time to access counselling and empowers them to let their needs to be known [3, 8, 23–26]. The success of the teen club shows the importance of a one-stop center with the availability of mentors and health providers to monitor and protect young people [3, 8, 23–26]. However, ALHIV in Malawi experience multiple challenges associated with their illness and various social, environmental, economic and cultural factors [3, 25]. Many ALHIV may be orphans, and may be managing concerns about food security, livelihood and household issues in addition to their health [3, 8, 25].

In 2015, Baylor College of Medicine International Pediatric AIDS Initiative (BIPAI) recommended HEADSS approach to screen the ALHIV that attend Teen Club (TC) programs in Malawi, a recommendation incorporated into the Malawian Ministry of Health Youth-Friendly Health Services Training Manual [3, 24, 26]. Despite this recommendation, ALHIV are not screened with HEADSS because of an increased number of ALHIV accessing Teen club in recent times. We therefore assessed the implementation barriers and facilitators of the HEADSS screening tool in Malawi.

### Conceptual/theoretical framework

We assessed barriers and facilitators to implementing HEADSS screening using semi-structured interview guides that contained questions based on the five domains of a Consolidated Framework for Implementation Research (CFIR) [27–32] (the intervention, inner setting, outer setting, the individuals involved, and the process by which implementation is accomplished) [27–32]. We selected constructs from the CFIR that were most relevant for this study. These five major domains as described in the CFIR [27–32] include:

1. Intervention characteristics which encompass seven sub-domains: the source of the innovation, the strength and quality of the evidence supporting it, the relative advantages the innovation provides, and its adaptability, trial-ability, complexity, design quality and packaging, and cost.
2. The outer setting entails the economic, political, and social contexts within which an organization resides. These may include constrained funding, billing rules, and the influence of policies related to treatment and targeted populations.
3. The inner setting comprises features of structural, political, and cultural contexts through which the implementation process will proceed.
4. Characteristics of the individuals involved with the innovation include their knowledge and beliefs about the intervention, self-efficacy, individual readiness to change, individual identification with the organization, and other personal attributes.
5. Implementation process encompasses the steps taken to introduce and sustain the innovation. The sub-domains entail planning; engaging opinion leaders, formally appointed internal implementation leaders, champions and external change agents; executing; and reflecting and evaluating [27–32].

We limited our selection to the 17 constructs that were relevant to our study aims. Our decision to select some of these constructs from the CFIR and not others was dictated by our core interest in assessing the KIs’ views regarding the how outer and inner setting characteristics affected the implementation of the HEADSS screening tool. We were also interested to know how they perceived the benefits of using this screening tool in their setting and whether the KIs found it user friendly or not. Additionally, we wanted to know their willingness to use this tool, the availability of resources to implement the HEADSS screening, and whether they felt that this tool can be integrated into the existing programs. Knowledge, skills, beliefs about HEADSS screening and self-efficacy were also assessed. Finally, we also wanted to find out how implementation of the HEADSS screening was planned with regard to training the health care providers (HCPs) and engagement of the management.

## Methods

### Study design

We conducted a cross-sectional descriptive qualitative study to assess the implementation barriers and facilitators of the HEADSS psychosocial screening in the two districts in southern Malawi.

### Study setting and population

The study took place in four TC program sites in Zomba and Machinga districts (one district hospital and one health center in each district). The sites were selected because they were implementing TC activities and involved a high number of active teen members and to represent urban and rural locations for greater transferability [33] of the results. The study population comprised of 20 key informants (KIs) who were drawn from, district health office (DHO), non-governmental organizations (NGOs) and public health facilities in selected Teen Clubs sites. It should be noted that many of the participants had not undergone training on the HEADSS screening. In addition, we did not proactively only include those respondents who had used the HEADSS because there is a lot of staff turnover in the health facilities in Malawi and it would be a challenge to find them.

### Sampling strategy and ethical procedures

A purposive sampling strategy was used to select the KIs in these four sites for a total of 20 research participants. Ethical approval for this study was obtained from the College of Medicine Research Ethics Committee (COMREC Ref No. P.01/19/2577). We also obtained written informed consents prior to the start of all interviews. All 20 participants who were approached consented to participate.

### Data collection and analysis

Data collection was coordinated and collected by the first author and one trained Research Assistant (RA) in social science and qualitative data collection. In each site in the two districts, five KIs were invited to take part in the semi-structured in-depth interviews, and this allowed us to identify and explore issues related to the potential barriers in the screening of psychosocial issues among ALHIV. Semi-structured in-depth interviews were audio-taped, transcribed verbatim and correlated with field notes to ensure that triangulation was achieved [34, 35]. In addition, the findings from several data sources such as different KIs especially from various organisations/institutions provided a more comprehensive understanding of the barriers and facilitators of HEADSS implementation and this also ensured the accuracy, reliability and balance of data collected [34, 35].

The transcripts of the interviews were entered into NVIVO 12 for analysis. The data were analyzed using thematic analysis [36, 37], inspired by a deductive directed approach, deemed applicable because we wanted to analyze our data in light of an existing framework. Coding and data analysis was done by the Principal Investigator. An initial list of thematic codes was generated from a subset of the transcripts based on the study's overall objectives and conceptual framework. Initial codes were created as nodes based on the 5 domains in the Consolidated Framework for Implementation Research (CFIR) framework and sub-nodes for the CFIR constructs [27–32]. These codes were refined through inductive analysis and results were organized according to identified key themes from the data. As the focus of this study was fairly narrow, namely, to look at barriers and facilitators of using HEADSS by applying specific constructs of the CFIR framework, we believed that the 20 KIIs were adequate and helped achieve data saturation [38, 39].

## Results

### Demographics

A total of 20 participants were interviewed from the four Teen Club sites in the two districts. These comprised of Adolescent District Coordinator (n = 1), District Psychosocial Counsellor (n = 1), District Technical Officer (n = 1), District Nursing Officer (n = 1) Clinical Officer (n = 1), Medical Technician (n = 1), Deputy Clinic Manager (n = 1), ART Nurse (1), Nurse & Midwife Technicians (n = 4), Monitoring and Evaluation Officer (n = 1) Midwife Technicians (n = 2), Psychosocial Support Mentor (1), Health Surveillance Assistants (HSAs) (n = 3) and HIV Diagnostic Assistant (HDA) (n = 1). The participants were predominantly males (n = 14/70%) and females (n = 6/30%). Their ages ranged from 25 to 49 years with mean age of 37 years. While a clinician provides specialized clinical care, a designated (non-physician such as the nurse, nursing aid, lay community health worker, expert client) staff member leads educational activities as well as crafts, sports and games. Clinicians have had training and mentorship in pediatric HIV care and nursing staff and lay health staff have been trained in the Teen Club Curriculum [4]. Even though all these cadres are called “Teen Club Mentors”, they have different role and responsibilities depending on their qualifications and expertise.

### Identification of barriers and facilitators using the Consolidated Framework for Implementation Research

We present the perceived barriers and facilitators according to the 5 domains and constructs of the CFIR framework.

#### Domain 1: Intervention characteristics

In this current study, five constructs were identified related to the intervention characteristics (e.g., intervention source, relative advantage, evidence strength and quality, complexity and design, quality and packaging). The following are the barriers and facilitators to implementation efforts.

##### Barriers to implementing the HEADSS screening

Regarding the participants’ perception about whether the HEADSS tool was externally developed, the majority had some reservations because the tool was not culturally specific to Malawi context, and they suggested that some culturally sensitive questions should be excluded from the tool. This was viewed as a barrier for the implementation:

*On the issues of these gender…. like lesbian, gay, bisexual, I think for these children I am not sure if we really need to ask these questions specifically to these children…..I am not sure myself but I have a fear to say if I pose these questions to these young ones, how do they take it…..? Like in Malawi, that’s not a culture, right. In Malawi these sexual issues such as bisexual, gay, lesbian, they are there but the community itself they don’t accept these* [ART Nurse Mentor].

*………they tool should include questions that are specifically for Malawi that are culture specific for example food security about Drugs domain, like what I am saying here in Malawi, people in the rural areas do not know much about coffee or tea, maybe they just hear about them but they do not know what they really are, they might have just heard about them in school that people drink coffee but they have not seen it themselves. Therefore, it would have been better if they had included say questions about breakfast in general, maybe asking if the children have had breakfast, the children would know that they are being asked about morning food. Or maybe probing if they do eat food in the morning because the adolescents sometimes look very depressed in the morning and it’s sometimes because they did not eat in the morning, they are hungry and weak…things like those* [Psychosocial Counsellor Mentor].

Participants also indicated that the screening tool was in English and some of the cadres that worked in TC program could not easily translate the contents into the local language (Chichewa). *This tool is in English and there are some who cannot read for instance and some of the things might be very difficult for them to translate* [Clinical Officer Mentor].

Additionally, the introduction of the HEADSS was not well communicated to the staff in these sites as the participants further explained that the engagement for HEADSS screening could have been increased if the implementation had been internally initiated from the Ministry of Health through the hospital administration as a mandatory intervention rather than an International Non-Governmental Organization (INGO):

*From my experience, it depends on who is introducing it. That is very important. If it somebody from outside and say this is it. It is difficult for them to accept it because they look at that individual like who is s/he. It's like if we don't do it, what is s/he going to do to us...you understand me. But let's say for example the one who is their direct supervisor of those health workers is the one who is bringing it and you develop a way of evaluating it then it can be done. That's mainly my experience. Because they say so …is my immediate supervisor, e.g here at this facility we have an ART coordinator or the Matron who acts as their coordinator, so if that tool the departmental head understands it well and if one brings it at the facility to the responsible staff, they get impressed and they regard it as important. For example, this tool is introduced by Ministry of Health, it will be respected and they will use it but if it comes haphazardly without proper channels, it will just die or disappear naturally. That's my experience* [ART Nurse Mentor].

##### Facilitators

The participants acknowledged that this psychosocial screening tool can guide better systematic counselling, build better client provider relationship, improve quality of care, and be good for holistic psychological assessment of ALHIV:

*Well, this (referring to HEADSS tool) is broad and it captures everything…..So with this one, we will make sure that we grasp every detail of them……..generally it is a good thing in a sense that it captures everything and it would help us specify what would be the needs for the adolescents living with HIV, secondly it would tell us what issues we should address in terms of managing them ……of psychological assessment* [ART Nurse Mentor].

Many of the participants further indicated that the HEADSS screening could fit into their existing work practice and was described as not very complex to implement. Most of the participants also perceived it as user-friendly, easier, clear, more trustworthy, and systematic. *To me, the design is just good and for somebody who is trained; it is user friendly* [HIV Diagnostic Assistant Mentor].

#### Domain 2: Outer setting

Three constructs were identified related to outer setting (e.g., patient needs and resources, external policies and incentives).

##### Barriers to implementing HEADSS screening

Some of the participants believed that ALHIV will be suspicious that the HCPs are policing on them when they ask them questions. They further expressed some concerns that the HEADSS screening might be time consuming for ALHIV:

*…adolescents might feel bored…. they will think that my friends are playing football, enjoying while this one is wasting my time now. So it has to be at least brief but with full information and not time consuming……..so it has to be tailor- made to our environment, considering the space,….. time, …..the numbers which we have and even considering that teen club is only half day clinic* [Medical Technician mentor].

Language was also reported as a barrier for some of the participants since the study was conducted in the two districts where Yao language is mainly dominated. As such, some of the HCPs were unable to communicate with the ALHIV.

*This is a Yao dominated area and it is a big challenge since this is the language for many of these adolescents…….it is very difficult because sometimes they do not understand us at all because they come from Yao origin and we are using Chichewa speaking people* [ART Nurse Mentor].

Incentives were mentioned as the biggest issue. HCPs might be willing to implement HEADSS screening if they were given some incentives in a form of cash since implementing the HEADSS would increase the workload because Teen Club program operates only on weekends so HEADSS implementation might be an additional task.

*Implementation of new things usually is accommodated when there is an incentive attached to it, from experience that’s what I have seen. If you just come and say we are adding to you this new tool to start conducting at your program or maybe at this facility HCPs will always assess if there are benefits attached to their needs especially incentives. But if you just bring a new tool without any incentive attached to the HCPs usually ignore that….* [Adolescent District Coordinator Mentor].

Limited social and economic resources were also identified by participants as a common challenge. They reported that most of the times, they provided health education on HIV and SRH issues, but mental/psychosocial health education was not incorporated in their program. The participants indicated that they felt if they provided psychosocial health education, the ALHIV might feel uncomfortable. The responses are not directly towards HEADDS screening barrier but to psychosocial issues in general in which HEADSS screening in part of this.

Yeah, the psychosocial services are very inadequate as already mentioned that we only give education on other things because if we were giving health education on psycho-social issues maybe the adolescents would have been comfortable to ask us some questions but if the adolescents see that you as providers are not talking about such a topic they might be afraid to ask yet they are able to ask some things because you have taught them about it [Nurse & Midwife Mentor].

*You know issues on mental health, young people are not accessing the services. I don't know whether it is due to the unavailability and inaccessibility……*[Medical Technician Mentor]

*Sometimes the adolescent has a psychosocial problem but since it is not a Teen Club day he or she will wait until it is Teen Club day on Saturday because the other days are adult clinic and the adolescents are so shy to come for clinic consultation* [Health Surveillance Assistant Mentor]

##### Facilitators

In general, all participants in all sites expressed a positive attitude toward the HEADSS screening tool and saw several potential advantages related to using it for screening ALHIV. They believed that HEADSS screening is essential in identifying and addressing many issues the ALHIV are facing in their lives:

*This tool is good because a lot of our adolescents are orphans. They are getting a lot of issues. They are depressed at home, at school …..the teen at home the support network is not that good. That teen will get depressed, but he cannot tell you that I'm meeting these issues at home. So, when you ask that teen using the HEADSS approach, you would possibly see that there are a lot of challenges that this teen is also meeting at home so when using this tool, you would possibly see the need of helping that teen* [ART Nurse Mentor].

#### Domain 3: Inner setting

Five constructs were identified related to inner setting (e.g., structural characteristics, networks and communications, culture, Implementation climate, leadership engagement).

##### Barriers to implementing the HEADSS screening

In all sites, participants expressed that there was no proper infrastructure or space to do the screening in privacy as one explained. *……. space is inadequate. The health providers would want to do some activities but where would they sit? The issues of HIV are sensitive, and you can't just sit anywhere?* [Health Surveillance Mentor].

….*maybe you have got 100 teens on that day like I have said about Z. Central and on that day maybe there are only 10 health care workers, for you maybe to assess maybe 20 teens it means you need more time and considering that even space is a challenge in most of health facilities. You cannot ask these questions while they are 50 teens* [ART Nurse Mentor].

*So, in some settings it is very difficult maybe because of no infrastructure that the facilities are using because for example provision of Teen Club in a Hall* [Health Surveillance Mentor].

The participants further expressed that there is a conservative culture toward implementation of new interventions. They described that HCPs liked to do things the way they always had done them:

*Implementation of something that is coming in as culturally a new thing, as I already explained, sometimes the health workers find it very difficult. Anything that is being introduced since we are already used to the old way of doing things so when we have a new initiative the health workers are not willing because they feel that this will delay them in their work. To some extent in the beginning, we have some issues until you go frequently to supervise them and monitor them until they internalize it* [Nurse Manager Mentor].

Furthermore, the participants reported that there was no proper orientation of HEADSS screening as one participant highlighted “*If this tool is introduced without proper orientation to the service providers, it would not be utilized accordingly resulting in a failure so it is important that it is introduced appropriately and that the service providers are knowledgeable about it then they can use it. As you have already noticed when you asked me I said I don't know this tool so if I would not have adequate time to learn what this tool is all about and you just give me to use it for sure it would not work because I would not know the importance of it but all in all the way it is and the information that is in this tool is very important and easy because it is not too much. Sometimes the information can be too much with just one important point but in this screening tool the key points are few and the ALHIV can fully benefit here* [District Nursing Officer Mentor].

##### Facilitators

TC Program was run by trained staff in all four sites. All participants were positive about the HEADSS psychosocial screening tool because they wanted to provide better services to ALHIV. Some of the participants indicated that workload issues might not be a challenge to screening of the ALHIV since they have different strategies to reduce workload such as task-shifting, SOP approaches for provider–client ratio:

*….there is a Standard Operation Procedure for a Teen Club of which there is a number of Psychosocial Providers which has to correlate to the ratio…to the number of the teens or adolescents that have to come on that day. So, the approach is those teens who have issues, their ratio with the providers have to tally. To say, a number of 5 teens or 10 teens have to be managed by one provider and another ten by another provider and even those teens who are suppressed (meaning viral load) the ratios have to be like that. So, I think like with 1:10 or 1:8 it is possible or manageable to implement HEADSS screening* [District Technical Officer Mentor]*.*

In addition, in some sites, availability of resources was perceived by some participants as sufficient in terms of staff to administer the HEADSS screening as well as the availability of the stationery as one participant explained: *“…….last year, Ministry (referring to MoH) including partners developed a minimum package for teen club. And if I remember very well, I think the minimum number of health care workers in a facility I think should be 5 or 6. So I consider that number…that one can be multitasked and can be assigned to do this role”* [Psychosocial Counsellor Mentor].

With regard to organizational commitment to its decision to implement the HEADSS screening, in terms of leadership engagement, in all sites it was found that leadership engagement at district level was described as strong and they were involved whenever there was a new initiative:

*As management we do support because when any new program comes, there is a management meeting, and we call general staff meeting explaining that we have this program…... Since management here receives programs well and it gives the program well to the service providers, the service providers also takes the initiative well and implement it. And when they implement it, they see that it gives good results. That is where the health care workers feel encouraged to continue* [Deputy Clinic Manager Mentor].

#### Domain 4: Characteristics of individuals

Two constructs were identified related to individual characteristics (e.g., knowledge and beliefs about the intervention and self-efficacy).

##### Barriers to implementing HEADSS screening

It was clear that issues of skills and competences were lacking. Knowledge and skills gap in HEADSS screening were reported as a barrier by most of the participants. Most of them did not have a training in mental health in general. The Teen Club mentors were mostly the Health Surveillance Assistants and HIV Diagnostic Assistants of whom most of them did not have any training in mental health and HEADSS screening.

*The major challenge is almost 90% if not all mentors are not trained in mental health that is specializing in adolescents as well as the HEADSS tool in screening psycho-social issues that might be affecting ALHIV that we are serving, so they don’t have the much expected expertise in identifying psychosocial issues that might be affecting adolescents that we are serving* [Medical Technician Mentor].

*…..As for challenges....inadequate knowledge and skills like even in HEADSS screening tool but also in psycho-social only few health workers got a 2-3 days training but if we look at the need it is big so it would be an opportunity if some are trained. In addition, currently we are depending on BCM who have brought their own professional psychosocial counsellor and this is a project and once it phases out, it means MDH will flop* [District Psychosocial Counsellor Mentor].

*Issues of psychosocial counselling, we tackle them at school as nurses and clinicians but though not in details but even here as a work station some service providers have been not trained or oriented on mental health* [ART Nurse Mentor]

*………I have never heard because if they had been trained ….maybe in combination of other training they have had that I might not know the content but not specific because we would have known since most of the times when there is a training on a certain topic we are informed as management. There is even a register at this office where we write that somebody has gone for this training but on this one, I have never heard that somebody has gone specifically for HEADSS screening tool training unless if it is something that was embedded in the training curriculum of something else, they were taught then I cannot know* [District Nursing Officer Mentor].

Some of the participants mostly the HCPs were of the opinion that screening the ALHIV will increase the workload and there was no time:

*I think it’s just to do with time, like I said earlier. For example today we had 105 teen club members who were here. And to say the screening tool to be used it can take this one at least five minutes. So out of 100 children we can spend almost, it’s about 5 hours, 3 or 4 hours, yeah. So, it’s time consuming but it’s necessary to be used* [Midwife Technician Mentor].

Although many of the participants had not seen or used this screening tool in their setting, some indicated that they used some of the questions but not following the sequence of the questions in the tool:

*Well, the HEADSS screening tool itself we don't use it but some of these questions we use them …… we just pick and ask them* [Health Surveillance Assistant Mentor]

Increased workload for the HCPs was also a potential barrier as perceived by some of the HCPs in the two sites while this was not a challenge in the other two sites. In all four sites the volume of ALHIV were more than 100 seen in one day and this was perceived as a challenge by some:

….*the workload is there because there are many adolescents in Teen Club and I feel that the best way is to add more staff so that there should be division of activities to reduce the workload otherwise we cannot avoid it* [Nurse & Midwife Mentor].

Negative attitude of HCPs also emerged as some of the barriers as one commented that some of the HCPs were egocentric to share the knowledge, sometimes they also had negative attitude towards the ALHIV*:*

*Health workers always doubt themselves, when providing care. Sometimes because of inadequate knowledge and skills and sometimes egocentric attitude because I might know that someone can provide this service better than myself because s/he is knowledgeable about this subject/topic, but we still want to do it ourselves and as such we provide poor quality care* [ART Nurse Mentor].

*We have inadequate knowledge and skills like even in HEADSS screening tool since is just new to us* [Monitoring and Evaluation Officer Mentor]

##### Facilitators

Regarding self-efficacy of the HCPs to administer the HEADSS tool to screen the ALHIV, some of the participants expressed that they were able to use it in their setting without any challenges, however; for those who had never used it they believed they would be able to use it if they were given a proper orientation as one said:

*I don’t struggle to use the tool because firstly when I arrive at the clinic, I am supposed to welcome the children and when I welcome those children, I make sure that all of them are used to me like their friend, they are not afraid of me, that’s the first. So, because the children are used to me, when I arrive, I manage to notice that this child is not how he normally is (that something is wrong with him or her). So, I isolate those ones and chat with them to find out what to do and while chatting with them using the HEADSS tool it’s when I realize there is something wrong with that child. And when that happens, I am supposed to take time with the child in a special way to see what is going on and what is the problem* [Psychosocial Support Mentor].

*As for me, I’m happy to use this tool because it guides me. Sometimes you do the counselling when your head is also blank, and you leave a lot of issues just because you do not have a guide so with the HEADSS tool it helps me to tackle all areas because without this I might forget other important issues and miss a lot of questions and at the end of the day I might not know other problems the adolescent is facing* [Nurse & Midwife Technicians Mentor].

*Yes, as long as I've been shown and trained then I will have the skills to be able to use it* [HIV Diagnostic Assistant Mentor].

*…….unless if we have gone for an orientation but I don't know is this tool just like this one or it's in a form of a flip-chart or …*[Health Surveillance Assistant].

#### Domain 5: Implementation process

Within the implementation process domain, two constructs were identified (e.g., planning and engaging).

##### Barriers to implementing HEADSS screening

With regard to integration of HEADSS screening tool in the TC program, the potential highlighted barrier as reported by several participants was that HEADSS screening could have had better uptake and acceptance if it had been introduced by government channels (MoH) rather than an NGO as some suggested:

*It should not be introduced in this facility by an NGO because that is where we miss the point and once the results of this research are out and you have given the recommendations and it is taken on board, it should not be introduced by an NGO because if it is being introduced by an NGO the health providers will consider it as an NGO thing. So, this should be incorporated into the main system of the government. The clinicians at government level need to incorporate this. At the end of the day and when they are appraisals they should be evaluated if this tool was utilized for example appraising them on how many clients were seen with some psychosocial issues using the HEADSS tool. How many clients were referred to psychosocial expert and how many were taken care using the skills in this regard? And if they do not have any skills, what did they do?* [Medical Technician Mentor].

*If there are initiatives regarding adolescents, they should not just be introduced just like that for example it should not be that D. or BCM or OC come to introduce their initiatives. I feel that in order to have the maximum impact, I feel that it has to be what the Ministry of Health want to introduce or implement. You will see that many initiatives are being brought by NGOs and they are the ones to evaluate their employees and government workers are being appraised by their superiors so even if the program would exist it cannot have the expected positive outcome at the end because government employees might not take ownership of such initiatives* [ART Nurse Mentor].

##### Facilitators

The participants expressed that all service providers should be able to use the HEADSS; it should be integrated into the existing programs; there should be briefing meetings to sensitize HCPs about HEADSS screening; HEADSS screening questions should be administered before ART refill.

*We need to sensitize the health workers if there can be a training or orient them…let’s train them, and then from there it’s when probably we can integrate the tool into our Teen Club services as well. But what I was thinking is that probably we can allocate someone so that that particular individual should be asking each and every teen who has come to our facility using this tool so that we can pick up the problems that our teens are encountering in their day-to-day life* [Nurse & Midwife Technicians Mentor].

Leadership engagement was described as strong and was involved whenever there was a new initiative in all sites.

*As management we do support because when any new program comes, there is a management meeting, and we call general staff meeting explaining that we have this program…... Since management here receives programs well and it gives the program to the service providers, the service providers also receive the initiative and implement it well. And when they implement it, they can see that it gives good results. That is where the health care workers feel encouraged to continue performing well* [Deputy Clinic Manager Mentor].

## Discussion

The current study identified important barriers and facilitators for the implementation of the HEADSS approach in adolescents ART program in some selected health facilities in Malawi from the HCPs’ perspective.

The main barriers identified within the intervention characteristics domain included that most of the participants were not officially informed that the HEADSS approach was recommended by MoH to be implemented during the routine ART delivery of the ALHIV. The HEADSS was introduced during their training, but that it was unclear if HCPs were to use HEADSS to screen the ALHIV. Additionally, the HEADSS was viewed as not culturally appropriate to Malawi context. Specifically, some questions on sexual orientation in the HEADSS tool were viewed as inappropriate. Our findings support previous studies which found that the lack of culturally appropriate mental health assessment instruments is a major barrier to screening individuals into mental health [40, 41]. Despite the above mentioned barriers, the participants were positive about HEADSS screening and they highlighted that it could be a good for holistic psychological assessment for ALHIV and also that it could fit into the existing work practice, it was user-friendly, easier, clear, more trustworthy, and systematic as facilitators.

Within the outer setting domain, Yao language, incentives and the lack of mental health education or psychosocial information given to ALHIV were noted as the main barriers. A study of South Africa found that healthcare workers complained about the lack of child-friendly job aides which they felt were essential in the process of effectively communicating with children through using age-appropriate language [41]. Language barriers within the health system could negatively affect ALHIV ART adherence levels. Studies have shown that patients who receive comprehensive information and who have access to culturally sensitive health services and who have open dialogue with their HCPs are more likely to be adherent to both ART regimens and clinical follow up [42–45].

A conservative culture towards implementation of new interventions, inadequate resources such as space and no proper orientation of the HEADSS screening tool were also reported as some of the challenge to screen the ALHIV within the inner setting domain. Clinical infrastructure has also been shown to be important in other clinical improvement efforts [41–45]. We found that the managers believed there were adequate resources such as human resources (enough staff) to implement HEADSS screening because MoH had developed a minimum package for Teen Club and also the implementation of multitasking would be desirable as a main facilitator in this domain. Leadership support and engagement are crucial for successful implementation and strategies toward the leaders should be included in the implementation plan [41, 42].

The identified barriers within the characteristics of individuals domain were HCPs knowledge and skills gaps because of HCPs not being trained in HEADSS screening and menta health, perceived work overload as well as time consuming from the perspectives of both HCPs and the ALHIV and HCPs negative attitudes towards the ALHIV. There is evidence suggesting that HCPs fail to provide HIV services to adolescents because they lack adequate knowledge and skills, and also lack of training on existing guidelines [40]. In addition, insufficient time tends to be a common barrier to successful implementation. These findings are supported by other studies [28, 31, 32, 43, 44] and these identified barriers as crucial for a successful implementation of the intervention are consistent to the barriers in previous studies [28, 31, 32, 42, 43]. Health system employees are already overburdened with effectively executing their jobs and adding another requirement might be viewed as a burden by many employees. However, it also showed that the HCPs believed they would be able to use the tool if they were given a proper orientation. Clinical practices are busy, and they may lack the capacity (due to limited staffing, time constraints, or frequent staff and leadership turnover) to implement an intervention that involves substantial effort on the part of clinical staff [43, 44].

Within the implementation process domain, the barriers identified included HCPs low willingness to implement a new initiative which is introduced by an NGO. However, the participants further indicated that the HEADSS screening could be integrated into the existing HIV services. Reviews of facilitators and barriers in health care settings show that while HCPs often understand the value of collaboration, they can be reluctant to engage when new ways of working clash with their existing professional experience [46, 47].

Common experiences of across low and high-income countries show that orienting policies, communications, and incentives to support and reward collaborative practice in clinical settings is essential [46, 47]. Before initiating a program, policymakers and program implementers need to establish clear policies and protocols on team member responsibilities; orientation and ongoing training for teamworking; regular patterns of communication and supervision that are institutionalized [46, 47].

Increased demand for healthcare services in countries experiencing high HIV disease burden and often coupled with a shortage of health workers, has necessitated task shifting from professional health workers to Lay Health Workers (LHWs) in order to improve healthcare delivery [48]. In Malawi, in addition to aspects of care related to other acute and chronic health conditions, treatment support for both TB and HIV has been task-shifted to LHW cadre established by the Malawi Ministry of Health, known as Health Surveillance Assistants (HSAs). Prior study in Malawi recommended that Teen Club mentors can assist with HEADSS screening prior to ALHIV seeing clinician then clinicians and nurses are commended to review HEADSS screening to allow for additional opportunities to discuss issues as well as discuss referral/treatment options (psychosocial counselor, social work, and nutritionist) [19]. However, there is a need to officially have an assigned cadre to perform the HEADSS screening. These findings highlight the crucial need to train the LHWs to administer the HEADSS since they mostly come from the same communities where they work, and they can speak the language. The role of local health care personnel is crucial, not only for communicating with the ALHIV in their own language, but also for understanding the mentality of the local population [49, 50]. Information should be provided in local languages and methods need to be found to supply patients continuously with updated and accurate information [49, 50].

We also recommend that non-monetary incentives such as professional development and performance-based incentives should be considered as evidence has shown that monetary incentives alone do not influence health worker performance [46]. In addition, adopting a proactive approach and addressing the incentives issues during program initiation would be the best strategy as it has shown to be effective in one study in Malawi [51].

## Strengths and limitations

The strengths of the study are the inclusion of different health care professions with some characteristics specific to the study aim, in light of their professions and experiences. We used an existing framework within the field of implementation science, CFIR, to better understand, describe, and identify factors that predict the likelihood of implementation success. Our possible limitations are that practically we could have selected the HCPs who were trained in the HEADSS approach and had been implementing/implemented it so that we could have been able to get their actual experiences of how well it has worked or did not work. In addition, our paper clearly presents only the HCP perspectives, it would have been better to include adolescents who had participated in the HEADSS screening to solicit the HEADSS screening acceptability or non-acceptability since this would have contributed to a more holistic understanding of the programme. However, the aims of this study were relatively narrow and precise.

## Conclusions

The key study findings showed that the HCPs perceived HEADSS tool as too long and time consuming and that it lacked appropriate cultural context. Systematic screening of ALHIV would allow the HCPs to identify mental health needs more clearly, indicating which ALHIV should receive mental health services, either further assessment or direct referral for treatment by a clinician. A screening tool more culturally appropriate to the Malawian context, especially one that can be used by non-clinicians such as lay health workers, would improve the ability to address mental health needs of ALHIV in many primary care and social service settings where resources for professional mental health staff are limited. This study will directly inform the efforts to cultural adaptation of the HEADSS screening tool for detection of mental and risky behaviors among ALHIV attending ART program in Malawi. Using the CFIR [44] to guide data collection, coding, analysis, and interpretation of findings supported a systematic comprehensive, and timely understanding of barriers and facilitators to implementation of the HEADSS psychosocial assessment tool for ALHIV in TC program.

## Data Availability

Data supporting the results are available from the authors upon request and with permission from College of Medicine in Malawi.

## Abbreviations

AIDS: Acquired Immune Deficiency Syndrome
ALHIV: Adolescents living with HIV
ART: Antiretroviral therapy
ARVs: Antiretroviral drugs
CFIR: Consolidated framework for implementation research
COMREC: College of Medicine Research Ethics Committee
DOT: Daily observed therapy
EBI: Evidence-based interventions
ED: Emergency department
FGDs: Focus group discussions
HCPs: Health care providers
HDA: HIV diagnostic assistant
HEADSS: Home, education and employment (eating and exercise), activities, drugs, sexuality, suicide/depression
HIV: Human Immuno-Deficiency Virus
HSA: Health Surveillance Assistant
INGO: International non-governmental organization
NAC: National AIDS commission
NGO: Non-governmental organization
RA: Research assistant
TC: Teen club
YLD: Years lost to disability
